# Towards implementation of rapid diagnostic tests for malaria case management in health centers of the Republic of Congo

**DOI:** 10.1186/1475-2875-11-S1-P71

**Published:** 2012-10-15

**Authors:** Francine Ntoumi, Jeannhey C Vouvoungui, Rod Ibara, Miguel Landry, Anissa Sidibé

**Affiliations:** 1 Congolese Foundation for Medical Research, Brazzaville, Republic of Congo

## 

Since the introduction of artemisin-based combination therapies in the Republic of Congo, limited number of investigations have been conducted to evaluate the burden of the disease at the district level. The main goal of this study was to document laboratory-confirmed cases using rapid diagnostic tests of malaria in children and pregnant women attending health facilities located in Northern districts of Brazzaville and Pointe Noire which is the second main city of the Republic of Congo. As objective2, the malaria diagnostic performance of each health facility has been assessed and as objective3, genetic diversity, multiplicity of infection and the prevalence of *P.falciparum* resistance gene markers have been investigated during the malaria transmission October 2011 to February 2012. More than 1500 children and 700 pregnant women were recruited. The prevalence of malaria was comprised between 9% and 30% depending of the localization of the health center. It was found that acceptability of technicians to use rapid diagnostic tests was low and microscopy is still considered as the reference. A comparison between health centers and reference lab technicians showed similar level of in malaria diagnosis performance. The *P.falciparum* genetic diversity (20 msp2 gene alleles) and multiplicity of infection (1.7) do not show any reduction but parasite densities were lower than reported in studies conducted before the introduction of ACTs. As a conclusion: in order to improve quality of care and the acceptability of RDT, there is a need to provide a targeted training to heath workers.

